# Association between hypnotic medication use and in-hospital falls among older adults: A multicenter landmark analysis

**DOI:** 10.1371/journal.pone.0351299

**Published:** 2026-06-08

**Authors:** Takuya Nishino, Yoshiaki Kubota, Yasuo Miyagi, Nari Tanabe, Fumiko Yamaguchi, Hiroki Ito, Shizuka Soh, Ayako Yano, Masako Mizuno, Chol Kim, Yosuke Ishii, Yukihiro Kondo, Kuniya Asai

**Affiliations:** 1 Department of Health Policy and Management, Nippon Medical School, Tokyo, Japan; 2 Department of Cardiovascular Medicine, Nippon Medical School, Tokyo, Japan; 3 Department of Medical Safety Control, Nippon Medical School Hospital, Tokyo, Japan; 4 Department of Cardiovascular Surgery, Nippon Medical School, Tokyo, Japan; 5 Faculty of Engineering, Suwa University of Science, Nagano, Japan; 6 Department of Medical Safety Control, Nippon Medical School, Chiba Hokusoh Hospital, Chiba, Japan; 7 Nursing Department, Nippon Medical School, Chiba Hokusoh Hospital, Chiba, Japan; 8 Department of Anesthesiology, Nippon Medical School, Chiba Hokusoh Hospital, Chiba, Japan; 9 Department of Urology, Nippon Medical School, Tokyo, Japan; University of Thessaly Faculty of Medicine: Panepistemio Thessalias Tmema Iatrikes, GREECE

## Abstract

**Background:**

Hypnotics are frequently prescribed to hospitalized older adults, but comparative evidence on fall risk across hypnotic classes remains inconsistent, partly due to confounding by indication and inadequate consideration of time at risk during hospitalization.

**Methods:**

We conducted a retrospective multicenter landmark analysis using administrative claims and electronic medical records from two acute-care hospitals in Japan (2018–2024). A day-7 landmark was defined, including patients aged ≥65 years who remained hospitalized and fall-free through hospital day 7. Sustained hypnotic exposure during hospital days 4–7 (≥2 days) was categorized as no sustained use, benzodiazepines/Z-drugs (BZ/Zs) alone, orexin receptor antagonists or ramelteon (ORA/Ram) alone, or combination therapy. The primary outcome was time to first in-hospital fall during hospital days 8–37. Cox proportional hazards models with multiple imputation were used. Competing-risk analyses and propensity score–matched comparisons between single-class users were conducted as sensitivity analyses.

**Results:**

Among 61,663 patients, 3,884 (6.3%) experienced an in-hospital fall after the landmark. Compared with no sustained hypnotic use, adjusted hazard ratios (HRs) for falls were 1.42 (95% CI, 1.24–1.64) for BZ/Zs, 1.46 (95% CI, 1.25–1.70) for ORA/Ram, and 1.42 (95% CI, 1.05–1.91) for combination therapy. Results were consistent in competing-risk analyses. In propensity score–matched analyses restricted to single-class users, fall risk did not differ significantly between BZ/Zs and ORA/Ram (adjusted HR, 0.98; 95% CI, 0.75–1.28).

**Conclusions:**

Sustained hypnotic use during hospitalization was associated with a higher incidence of in-hospital falls among older adults. After adjustment for measured clinical factors and treatment selection, no significant difference in fall risk was observed between BZ/Zs and ORA/Ram.

## Introduction

In-hospital falls are a frequent and serious adverse event among older adults and represent a major geriatric syndrome associated with fractures, functional decline, prolonged hospitalization, institutionalization, and increased mortality [1,2]. In acute care hospitals, fall risk is particularly high because many older patients are admitted emergently and experience impaired consciousness, reduced physical function, or intensive care unit stays [3]. This heightened risk reflects the multifactorial nature of falls in hospitalized patients, as shown in recent large-scale, multicenter analyses integrating clinical, functional, and medication-related factors. Importantly, fall risk during hospitalization is not a static state but evolves over time, underscoring the need for continuous reassessment and appropriate clinical management [4]. Sleep disturbances are ubiquitous among hospitalized older adults, making the selection of hypnotic medications a critical component of fall prevention strategies [5,6]. Benzodiazepines and Z-drugs (BZ/Zs), which have been widely prescribed for decades, are well known to increase fall risk through sedative and muscle-relaxant effects [7,8]. In contrast, physiologic sleep-promoting agents, such as ORA/Ram, which promote sleep by selectively suppressing the wake-promoting orexin system, have been increasingly used as alternative agents because of their more physiologic sleep profile [9,10]. However, evidence regarding the association between ORA/Ram use and in-hospital falls remains inconsistent, with observational studies reporting reduced, neutral, or even increased fall risk [11–13].

A key explanation for these conflicting findings is confounding by indication [14]. In real-world clinical practice, clinicians tend to avoid BZ/Zs and preferentially prescribe ORA/Ram to patients perceived to be at higher risk of falls, such as those with impaired consciousness or poor physical function [15]. If this prescribing pattern is not adequately accounted for, the observed association between ORA/Ram use and falls may reflect underlying patient vulnerability rather than a true drug-related effect. Despite its clinical relevance, few studies have quantitatively addressed this issue in high-risk hospitalized older populations, particularly in acute care. Furthermore, most prior studies have evaluated hypnotic safety without sufficiently accounting for the dynamic nature of hospitalization. Patients with longer hospital stays are exposed to hypnotics for longer periods and have more opportunities to experience falls, which may bias comparisons if exposure duration is not properly considered. These methodological limitations may partially explain the divergent conclusions in the existing literature.

To address these gaps, we evaluated the association between sustained hypnotic use during hospitalization and subsequent in-hospital falls among older adults with prolonged hospital stays, using a framework that reflects real-world prescribing decisions. We compared fall risk across four exposure groups and directly contrasted BZ/Zs with ORA/Ram after rigorous adjustment for clinical characteristics. By including high-risk patients often excluded from clinical trials, this study aims to clarify the safety profile of ORA/Ram and provide practical evidence to support rational hypnotic selection and benzodiazepine deprescribing in acute geriatric care.

## Methods

### Study design and setting

This retrospective observational multicenter study was conducted using inpatient data from two acute-care hospitals in Japan: Nippon Medical School Hospital and Chiba Hokusoh Hospital. The dataset was compiled from the Diagnosis Procedure Combination (DPC) data and laboratory test results extracted from electronic medical records. Ethics approval for this study was obtained from the central ethics review committee of Nippon Medical School (approval M-2023–137). This study adhered to the principles of the Declaration of Helsinki. Informed consent was obtained using an opt-out approach. This study is reported in accordance with the STROBE guidelines for observational studies. Study data were accessed for research purposes from November 1, 2025 to November 30, 2025. The authors analyzed de-identified data only and did not have access to information that could identify individual participants during or after data collection.

### Patients and landmark

All patients included in this study were hospitalized at the study facility between April 2018 and March 2024. Patients younger than 65 years were excluded. A landmark analysis was conducted on hospital day 7 (LM7), including patients who remained hospitalized and fall-free through hospital day 7. Patients who died, were discharged, or experienced a fall before hospital day 7 were excluded from the risk set. Patients who died after the landmark day without experiencing a fall were retained in the analysis and censored at the date of death. The choice of hospital day 7 as the landmark was based on two main considerations. First, it allowed for a sufficient exposure assessment window (hospital days 4–7) to define sustained hypnotic use and minimize exposure misclassification. Second, it reduced the influence of acute clinical instability immediately after admission, during which both prescribing decisions and fall risk may be highly variable. This approach was intended to better reflect stable prescribing patterns and subsequent fall risk in routine clinical practice and is consistent with prior observational studies using landmark designs to reduce bias related to early clinical instability.

### Outcomes

The primary outcome was the first documented in-hospital fall occurring within 30 days after the landmark day (hospital days 8–37). A fall was defined as an unintentional descent to the floor or another lower level, regardless of injury. Reporting within this system is compulsory for all fall events and is conducted by healthcare staff as part of routine patient safety monitoring, and includes detailed contextual information such as the time, location, circumstances, and severity classification of each incident, ensuring high completeness and reliability of outcome ascertainment. Although this system is designed to capture a wide range of fall events, minor or unwitnessed falls may be underreported.

### Variables

Variables were defined based on information available at LM7, unless otherwise specified. Time-invariant baseline variables included age and sex. Clinical information was obtained from the DPC database, including demographics, comorbidities, procedures, and medication records. Activities of daily living (ADL) were assessed using the institutional nursing care needs score. This score evaluates the need for assistance with basic activities of daily living, the ability to comprehend medical instructions, and the presence of risky behaviors (Supplementary S1 Table). Severity of illness was additionally characterized using the cumulative number of days spent in the intensive care unit (ICU) prior to the LM7 landmark.

Laboratory data were extracted from electronic medical records to capture physiological factors potentially associated with falls, including nutritional status, inflammation, renal function, and electrolyte balance. For each laboratory parameter, the LM7 value was defined as the measurement closest to hospital day 7 within a ± 1-day window (hospital days 6–8). Use of medications other than sleep medications was evaluated according to the institutional fall risk assessment protocol and included oral steroids, diuretics, antiparkinsonian drugs, psychotropic medications, and antidiabetic drugs. Exposure to these medications was defined as at least one administration or prescription during hospital days 4–7 and was included as covariates in the analysis.

Sleep medication exposure, the primary exposure of interest, was determined based on prescription records during the 4-day pre-landmark window between hospital days 4 and 7. To capture the effects of sustained pharmacological treatment and minimize the influence of sporadic or single-dose administration—such as *pro re nata* (as-needed) use—exposure was defined as the administration of a specific drug class for two or more days within this period. Following this definition, patients were categorized into four mutually exclusive groups: the **control group (A)** comprised patients who received no sleep medications or used them on only a single day during the exposure window; the **pure BZ/Zs group (B)** included those who received benzodiazepines or Z-drugs for at least two days without concomitant ORA/Ram use; the **pure ORA/Ram group (C)** consisted of patients receiving ORA/Ram for at least two days without BZ/Zs co-administration; and the **combination group (D)** was defined as patients who received both BZ/Zs and ORA/Ram for two or more days each.

### Statistical analysis

Baseline characteristics were summarized using descriptive statistics, with categorical variables presented as counts (percentages) and continuous variables presented as medians with interquartile ranges.

The primary analysis evaluated the association between sleep medication exposure and time to first in-hospital fall after the LM7 landmark using Cox proportional hazards models. The LM7 cohort comprised patients who remained hospitalized and fall-free through hospital day 7. Follow-up started on hospital day 8 and continued for 30 days (through hospital day 37), with time indexed as days since hospital day 8. Falls occurring within this period were defined as the event of interest. Patients who were discharged without experiencing a fall were censored at the date of discharge, and those who died without a fall were censored at the date of death. A time-to-event framework was adopted to account for variation in length of hospital stay and differential exposure opportunity, which cannot be adequately addressed using binary outcome models. Multivariable Cox proportional hazards models were fitted to compare fall risk across four mutually exclusive sleep medication exposure groups, with the control group serving as the reference category. Models were adjusted for age, sex, body mass index, activities of daily living at LM7, laboratory values at LM7, and concomitant medications included in the institutional fall risk assessment protocol. Missing covariate data were handled using multiple imputation by chained equations (m = 20). We considered the missingness mechanism to be compatible with missing at random, because missing laboratory data at the Day 7 landmark were thought to mainly reflect observed clinical condition and routine testing practices rather than the unobserved values themselves. The imputation model included exposure group, event indicator, follow-up time, and all covariates used in the analysis. The time-to-event variable, event indicator, and exposure group were not imputed. Cox models were fitted separately within each imputed dataset, and hazard ratio estimates were pooled using Rubin’s rules. The proportional hazards assumption was assessed using Schoenfeld residuals within each imputed dataset, and p-values were summarized across imputations to evaluate the consistency of the assumption. Kaplan–Meier curves were generated to visualize the cumulative incidence of in-hospital falls, expressed as 1 − S(t), across exposure groups in the unadjusted cohort, and between-group differences were assessed using the log-rank test.

As sensitivity analyses, we assessed the robustness of the primary findings using Fine–Gray subdistribution hazard models, treating falls as the event of interest. First, in-hospital death was treated as the competing event. Second, an additional competing-risk analysis was performed treating both in-hospital death and hospital discharge as competing events. Fine–Gray models were fitted in a complete-case dataset aligned to the LM7 time axis. Results were reported as subdistribution hazard ratios with 95% confidence intervals. In an additional sensitivity analysis, the nursing care needs score at LM7 was modeled as a categorical variable (Low [score = 0], Middle [score = 1–4], and High [score ≥5]) to account for potential non-linearity. The components and scoring of the institutional nursing care needs score are provided in Supplementary S1 Table.

After multiple imputation, propensity score estimation, matching, and effect estimation were performed within each imputed dataset, and estimates were combined using Rubin’s rules. As a secondary analysis to directly compare medication safety, the cohort was restricted to patients receiving BZ/Zs alone or ORA/Ram alone. Within each imputed dataset, propensity scores were estimated using logistic regression models incorporating baseline characteristics and LM7-aligned covariates. One-to-one nearest-neighbor matching without replacement was performed within each dataset using a caliper width of 0.2 standard deviations of the logit-transformed propensity score. Covariate balance before and after matching was evaluated using standardized mean differences (SMDs), with values <0.1 indicating adequate balance. In the matched cohorts, Cox proportional hazards models were fitted using MatchIt-derived matching weights, with robust standard errors clustered by matched set (subclass). Prespecified subgroup analyses were conducted within the post-matching cohorts across clinically relevant strata to assess the consistency of the association between BZ/Zs and ORA/Ram.

All statistical tests were two-sided, and p values < 0.05 were considered statistically significant. Statistical analyses were performed using R software.

## Results

### Study population and baseline characteristics

**Fig 1:**
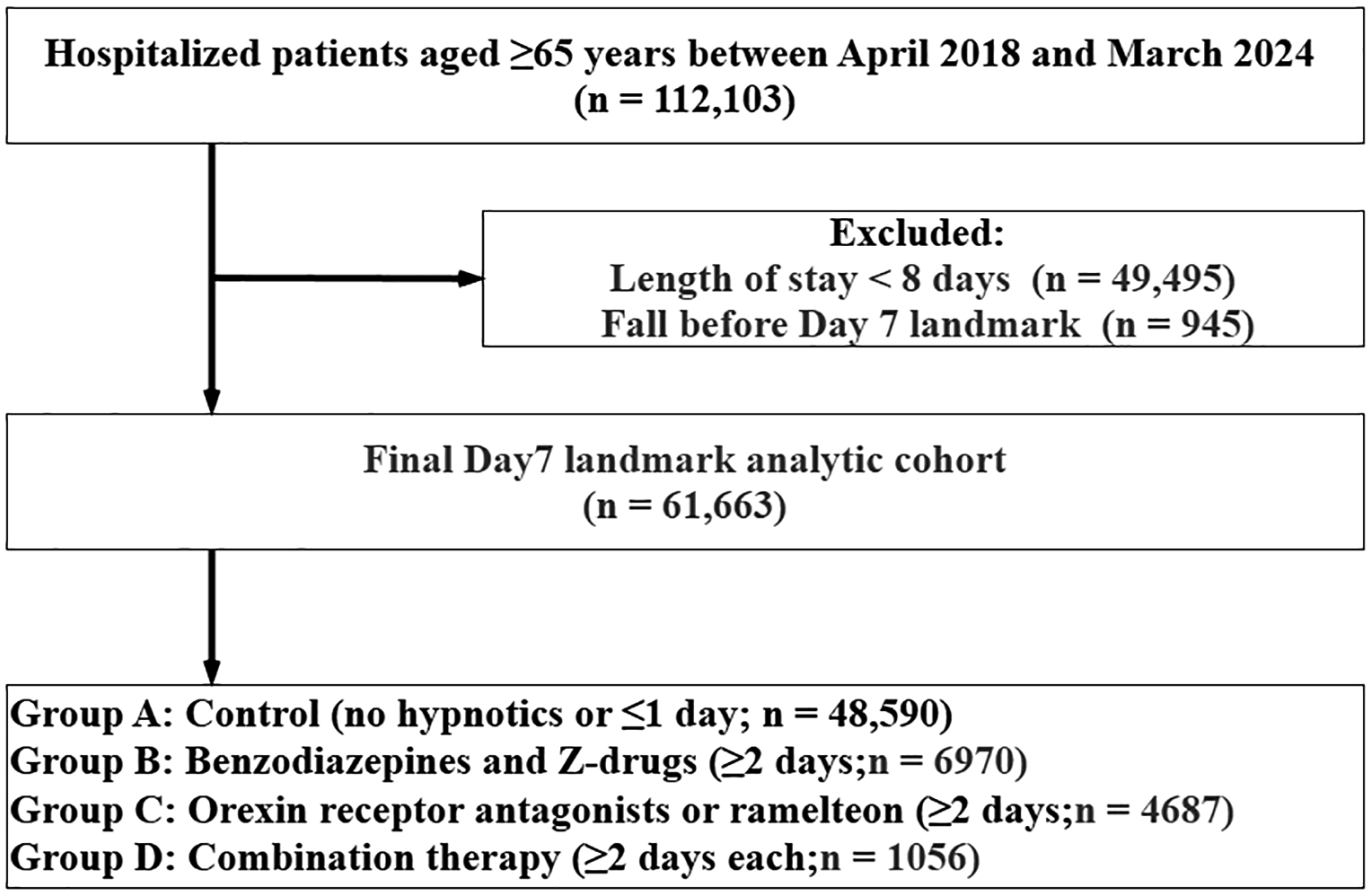
Study flow diagram and cohort definition. Flow diagram illustrating patient selection and construction of the Day-7 landmark analytic cohort. Among 112,103 hospitalized patients aged ≥65 years between April 2018 and March 2024, patients with a length of stay shorter than 8 days or who experienced a fall before hospital day 7 were excluded. The final Day-7 landmark cohort consisted of 61,663 patients who remained hospitalized and fall-free through hospital day 7. Based on sleep medication exposure during hospital days 4–7, patients were classified into four groups: control (no hypnotics or use for ≤1 day), benzodiazepines/Z-drugs only (≥2 days), orexin receptor antagonists or ramelteon only (≥2 days), and combination therapy (≥2 days each).

A total of 112,103 hospitalized patients aged 65 years or older between April 2018 and March 2024 were initially identified (Fig 1). Of these, 49,495 patients with a length of stay shorter than 8 days and 945 patients who experienced a fall before the day-7 landmark were excluded. The remaining 61,663 patients constituted the final Day-7 landmark analytic cohort. According to sleep medication exposure during hospital days 4–7, patients were classified into four mutually exclusive groups. Group A included 48,590 patients who received no hypnotic medications or used them for only one day. Group B comprised 6,970 patients who received benzodiazepines or Z-drugs for at least two days. Group C included 4,687 patients who received ORA/Ram for at least two days. Group D consisted of 1,056 patients who received both BZ/Zs and ORA/Ram for at least two days each. Zolpidem tartrate was the most frequently prescribed agent in the BZ/Zs group, whereas lemborexant, ramelteon, and suvorexant were most common in the ORA/Ram group (Supplementary S2 Table).

Baseline characteristics of the Day-7 landmark cohort stratified by sleep medication exposure are summarized in Table 1. Patients in Groups C and D tended to be older and were more frequently admitted emergently than those in the control group. Functional impairment, as reflected by the nursing care needs score at Day 7, was substantially greater in Groups C and D than in Groups A and B. In addition, Group C exhibited higher levels of inflammatory markers and renal dysfunction than the other groups, with higher median C-reactive protein and serum creatinine levels. Regarding early in-hospital treatments during days 4–7, the use of diuretics, psychotropic drugs, and rehabilitation was more common in Groups C and D than in the other groups, whereas patients in Group B generally showed values between those of Group A and Groups C/D across most clinical and treatment-related variables. ICU stay prior to the landmark was short in most patients, and the prevalence of malignancy differed across exposure groups. A substantial proportion of laboratory measurements at Day 7 were missing, reflecting routine clinical practice in which laboratory testing is often omitted in clinically stable patients; these missing data were subsequently addressed using multiple imputation in the adjusted analyses. SMDs indicated imbalances in several baseline characteristics between groups prior to adjustment, particularly for clinical severity and concomitant medication use.

**Table 1:** Baseline characteristics of the study population according to sleep medication exposure groups.

Variable	Overall (n = 61,663)	Group A (n = 48,950)	Group B (n = 6,970)	Group C (n = 4,687)	Group D (n = 1,056)	SMD A vs B	SMD A vs C	SMD A vs D	Missing, n (%)
Age, years	76 [71, 82]	76 [71, 81]	76 [71, 82]	79 [74, 85]	77 [72, 83]	0.056	0.392	0.158	—
Male sex, n (%)	36,459 (59.1)	29,706 (60.7)	3,466 (49.7)	2,731 (58.3)	556 (52.7)	0.220	0.049	0.162	—
Emergency admission, n (%)	31,542 (51.2)	24,481 (50.0)	3,108 (44.6)	3,391 (72.3)	562 (53.2)	0.109	0.458	0.064	—
Body mass index, kg/m²	22.3 [19.9, 24.8]	22.4 [19.9, 24.9]	22.1 [19.7, 24.6]	22.0 [19.5, 24.3]	22.1 [19.8, 24.7]	0.057	0.107	0.038	2,839 (4.6)
ICU stay, days	0 [0, 0]	0 [0, 0]	0 [0, 0]	0 [0, 3]	0 [0, 0]	0.241	0.402	0.060	
Malignancy, n (%)	24,146 (39.2)	19,685 (40.2)	2,939 (42.2)	1,152 (24.6)	370 (35.0)	0.040	0.334	0.107	—
Nursing care needs score at Day 7	2 [0, 5]	1 [0, 5]	1 [0, 4]	5 [2, 8]	3 [0, 6]	0.170	0.699	0.201	—
Day 7 laboratory values									
Serum albumin, g/dL	3.0 [2.6, 3.4]	3.0 [2.5, 3.4]	3.1 [2.7, 3.5]	2.9 [2.5, 3.3]	3.1 [2.7, 3.4]	0.177	0.159	0.125	25,192 (40.9)
Blood urea nitrogen, mg/dL	16.2 [11.6, 24.0]	16.0 [11.4, 23.7]	15.5 [11.0, 22.1]	19.1 [13.0, 28.4]	16.8 [11.1, 24.7]	0.067	0.234	0.065	22,057 (35.8)
C-reactive protein, mg/dL	2.39 [0.65, 6.46]	2.44 [0.65, 6.57]	1.87 [0.49, 5.42]	2.67 [0.88, 6.83]	2.02 [0.59, 5.57]	0.14	0.028	0.064	23,054 (37.4)
Serum creatinine, mg/dL	0.82 [0.63, 1.12]	0.81 [0.63, 1.10]	0.80 [0.63, 1.12]	0.88 [0.66, 1.27]	0.84 [0.64, 1.22]	0.043	0.096	0.049	22,016 (35.7)
Hemoglobin, g/dL	11.0 [9.6, 12.5]	11.0 [9.6, 12.5]	10.9 [9.5, 12.3]	10.8 [9.5, 12.2]	10.9 [9.7, 12.2]	0.091	0.091	0.086	21,809 (35.4)
Serum potassium, mmol/L	4.1 [3.7, 4.4]	4.1 [3.7, 4.4]	4.1 [3.7, 4.4]	4.1 [3.7, 4.4]	4.1 [3.7, 4.4]	0.022	0.009	0.002	22,123 (35.9)
Serum sodium, mmol/L	140 [137, 142]	140 [137, 142]	140 [137, 142]	140 [137, 142]	140 [137, 142]	0.091	0.014	0.117	22,122 (35.9)
White blood cell count, × 10⁹/L	6.6 [5.0, 8.9]	6.7 [5.0, 8.9]	6.2 [4.7, 8.2]	7.0 [5.4, 9.2]	6.3 [4.9, 8.6]	0.106	0.043	0.025	21,793 (35.3)
Early in-hospital treatments (days 4–7)									
Oral steroids, n (%)	5,683 (9.2)	4,349 (8.9)	780 (11.2)	429 (9.2)	125 (11.8)	0.077	0.009	0.097	—
Diuretics, n (%)	14,288 (23.2)	10,593 (21.6)	1,649 (23.7)	1,727 (36.8)	319 (30.2)	0.048	0.334	0.196	—
Antiparkinsonian drugs, n (%)	1,224 (2.0)	750 (1.5)	264 (3.8)	141 (3.0)	69 (6.5)	0.140	0.099	0.254	—
Psychotropic drugs, n (%)	6,629 (10.8)	3,489 (7.1)	1,277 (18.3)	1,482 (31.6)	381 (36.1)	0.336	0.620	0.704	—
Antidiabetic drugs, n (%)	16,729 (27.1)	13,355 (27.3)	1,630 (23.4)	1,456 (31.1)	288 (27.3)	0.090	0.083	0.000	—
General anesthesia during, n (%)	4,008 (6.5)	3,326 (6.8)	443 (6.4)	189 (4.0)	50 (4.7)	0.018	0.122	0.088	—
Rehabilitation during hospitalization, n (%)	15,282 (24.8)	11,765 (24.0)	1,488 (21.3)	1,720 (36.7)	309 (29.3)	0.064	0.275	0.118	—
Maintenance dialysis, n (%)	1,711 (2.8)	1,256 (2.6)	258 (3.7)	158 (3.4)	39 (3.7)	0.065	0.047	0.065	—

Values are presented as median [interquartile range] or number (percentage).Groups were defined based on sleep medication exposure during hospital days 4–7, as described in the Methods.

ICU stay represents cumulative days prior to Day 7.

Laboratory values correspond to measurements closest to Day 7 (±1 day). Values are rounded to one decimal place.

Standardized mean differences (SMDs) were calculated for pairwise comparisons using Group A as the reference group.

**Abbreviations:** ICU, intensive care unit.

### Primary analysis

**Fig 2:**
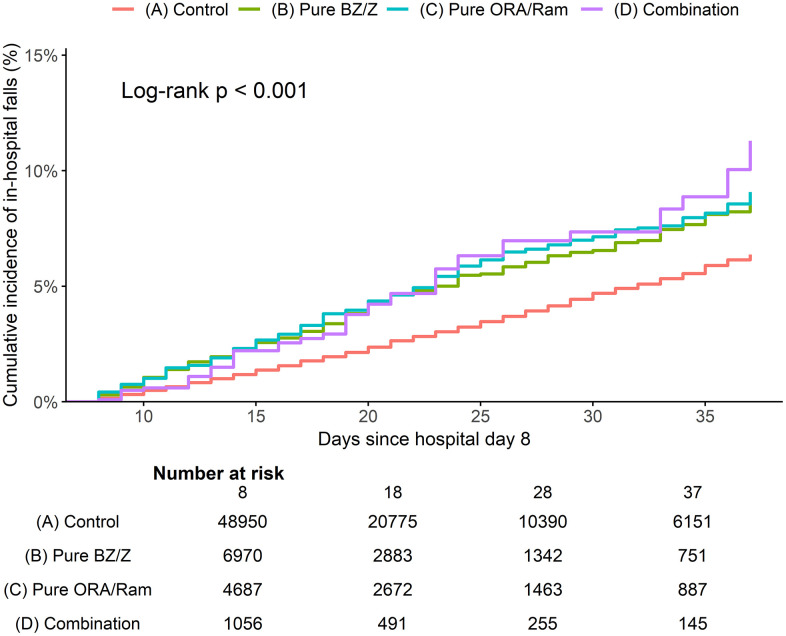
Kaplan–Meier curves for in-hospital falls after the Day-7 landmark. Kaplan–Meier curves show the cumulative incidence of first in-hospital falls (1 − S(t)) from hospital day 8 to day 37, stratified by sleep medication exposure during hospital days 4–7. Patients were categorized into four mutually exclusive groups: (A) control (no hypnotics or use for ≤1 day; n = 48,590), (B) benzodiazepines/Z-drugs only (≥2 days; n = 6,970), (C) orexin receptor antagonists or ramelteon only (≥2 days; n = 4,687), and (D) combination therapy (≥2 days each; n = 1,056). Numbers at risk are shown below the plot at selected time points. Between-group differences were assessed using the log-rank test. Note: These curves are based on Kaplan–Meier estimation with censoring; competing risks of in-hospital death and hospital discharge were evaluated in sensitivity analyses using Fine–Gray models.

Table 2 presents the results of Cox proportional hazards analyses evaluating the association between sleep medication exposure and time to first in-hospital fall after the LM7. In univariable analyses, all three hypnotic exposure groups showed significantly higher fall risk compared with the control group. The hazard ratio (HR) was 1.52 (95% CI, 1.33–1.75) for BZ/Zs alone, 1.74 (95% CI, 1.51–2.00) for ORA/Ram alone, and 1.67 (95% CI, 1.24–2.24) for combination therapy. Kaplan–Meier curves illustrating unadjusted cumulative incidence from LM7 onward are shown in Fig 2. Patients receiving BZ/Zs, ORA/Ram, or combination therapy exhibited a consistently higher cumulative incidence of in-hospital falls than the control group throughout follow-up. Separation of the curves became apparent shortly after LM7, and overall differences among groups were statistically significant by the log-rank test. After adjustment for demographic characteristics, functional status, laboratory variables, and concomitant medications using multiple imputation, these associations remained statistically significant. Compared with the control group, the adjusted HR was 1.42 (95% CI, 1.24–1.64) for BZ/Zs, 1.46 (95% CI, 1.25–1.70) for ORA/Ram, and 1.42 (95% CI, 1.05–1.91) for combination therapy. Among covariates included in the multivariable model at LM7, older age, male sex, malignancy, lower body mass index, lower serum albumin, lower hemoglobin, and lower serum sodium levels were independently associated with a higher risk of falls. In addition, use of oral steroids, diuretics, psychotropic drugs, and antidiabetic medications during hospital days 4–7 was associated with increased fall risk, whereas emergency admission and general anesthesia were not significantly associated with falls. The absolute risk of falls was 2.3% in the control group, 3.5% in the BZ/Z group, 4.9% in the ORA/Ram group, and 4.4% in the combination group (Table 3), corresponding to absolute risk differences of approximately 1%–3%. Although these associations were statistically significant, the absolute risk differences across exposure groups were modest. Assessment of Schoenfeld residuals suggested some deviation from the proportional hazards assumption. In the multiple-imputation analyses, evidence of non-proportionality was observed for the exposure group and in the global test across imputed datasets. However, given the relatively short 30-day follow-up period and the consistent direction of results across sensitivity analyses, hazard ratios from the Cox model were interpreted as average associations over the follow-up period.

**Table 2:** Association between sleep medication exposure and in-hospital falls after the Day 7 landmark.

Variable	Univariable HR (95% CI)	p value	Multivariable HR (95% CI)	p value
Sleep medication exposure				
BZ/Zs only vs control	1.522 (1.326–1.746)	<0.001	1.423 (1.236–1.639)	<0.001
ORA/Ram only vs control	1.735 (1.505–2.000)	<0.001	1.459 (1.254–1.698)	<0.001
Combination therapy vs control	1.667 (1.241–2.238)	0.001	1.416 (1.049–1.911)	0.023
Covariates (Day 7 unless noted)				
Age (per year)	—	—	1.009 (1.002–1.016)	0.017
Male sex	—	—	1.187 (1.069–1.318)	0.001
Emergency admission	—	—	0.981 (0.875–1.099)	0.737
Body mass index	—	—	0.973 (0.960–0.987)	<0.001
Nursing care needs score	—	—	1.000 (0.980–1.019)	0.96
Serum albumin (g/dL)	—	—	0.892 (0.797–0.999)	0.048
Serum creatinine (mg/dL)	—	—	1.029 (0.994–1.065)	0.104
Hemoglobin (g/dL)	—	—	0.958 (0.925–0.991)	0.015
Serum sodium (mmol/L)	—	—	0.961 (0.948–0.974)	<0.001
Malignancy	—	—	1.244 (1.109–1.395)	<0.001
ICU stay (days)	—	—	0.953 (0.928–0.979)	<0.001
Oral steroids	—	—	1.188 (1.025–1.377)	0.022
Diuretics	—	—	1.146 (1.024–1.282)	0.018
Antiparkinsonian drugs	—	—	1.255 (0.947–1.665)	0.114
Psychotropic drugs	—	—	1.631 (1.436–1.852)	<0.001
Antidiabetic drugs	—	—	1.125 (1.007–1.256)	0.037
General anesthesia	—	—	0.923 (0.756–1.128)	0.434

Hazard ratios (HRs) were estimated using Cox proportional hazards models. Multivariable models were adjusted for all covariates listed and fitted using multiple imputation (m = 20). Control indicates no sustained hypnotic use (≤1 day during hospital days 4–7). ICU stay represents cumulative days prior to Day 7.

**Table 3:** Absolute risk of in-hospital falls by sleep medication exposure.

Exposure group	Events / N (%)	Absolute risk (%)	Absolute risk difference (95% CI)
Control (A)	1,138 / 48,950	2.32	Reference
BZ/Z only (B)	247 / 6,970	3.54	+1.22 (0.76 to 1.67)
ORA/Ram only (C)	228 / 4,687	4.86	+2.54 (1.91 to 3.17)
Combination (D)	46 / 1,056	4.36	+2.03 (0.79 to 3.27)

In the complete-case Cox analysis (Supplementary S3 Table), all hypnotic exposure groups remained significantly associated with an increased risk of in-hospital falls compared with the control group, with effect estimates comparable to those in the primary analysis. In Fine–Gray subdistribution hazard analyses treating in-hospital death as a competing event (Supplementary S4 Table), the results were consistent with those of the primary analysis. In additional Fine–Gray subdistribution hazard analyses treating both in-hospital death and hospital discharge as competing events (Supplementary S5 Table), the direction and overall pattern of associations remained broadly consistent with those of the primary Cox model. In an additional sensitivity analysis, modeling the nursing care needs score as a categorical variable (Supplementary S6 Table) did not materially change the results.

### Secondary analysis

To minimize confounding by indication and directly compare hypnotic class–specific risk, we conducted a propensity score–matched analysis restricted to single-class users. Propensity score matching at the LM7 landmark resulted in a substantial improvement in covariate balance, with most standardized mean differences reduced below 0.1 (Supplementary S1 Fig). Before matching, several baseline and LM7-aligned covariates—including age, nursing care needs score, ICU length of stay, serum albumin level, emergency admission, malignancy, and concomitant use of psychotropic drugs—showed marked imbalance between groups, with SMDs exceeding 0.100. After matching, SMDs were reduced to below 0.100 for most covariates, indicating a marked overall improvement in balance, although a small residual imbalance slightly above this threshold remained for the nursing care needs score. Consistent with these findings, the distributions of propensity scores demonstrated substantially greater overlap after matching (Supplementary S2 Fig). Prior to matching, the propensity score distributions of the BZ/Zs and ORA/Ram groups were clearly separated, whereas after matching, the distributions largely overlapped, indicating improved comparability between groups. In Cox proportional hazards analyses directly comparing the BZ/Zs and ORA/Ram groups (Table 4), no significant difference in fall risk was observed either before or after matching. In the pre-matching cohort, the hazard ratio for falls in the BZ/Zs group relative to the ORA/Ram group was 0.950 (95% CI, 0.793–1.139) in the unadjusted model and 0.946 (95% CI, 0.771–1.161) after multivariable adjustment. In the matched cohort, the corresponding hazard ratios were 0.990 (95% CI, 0.758–1.295) in the unadjusted analysis with robust standard errors and 0.978 (95% CI, 0.749–1.277) after adjustment.

**Table 4:** Association between benzodiazepines/Z-drugs and in-hospital falls compared with orexin receptor antagonists or ramelteon.

Model	Hazard ratio (95% CI)	p value
Cox, pre-PSM (unadjusted)	0.950 (0.793–1.139)	0.57
Cox, pre-PSM (adjusted)	0.946 (0.771–1.161)	0.60
Cox, post-PSM (unadjusted, robust SE)	0.990 (0.758–1.295)	0.95
Cox, post-PSM (adjusted, robust SE)	0.978 (0.749–1.277)	0.88

Hazard ratios were estimated using Cox proportional hazards models. Propensity score matching (PSM) was performed using 1:1 nearest-neighbor matching without replacement. Post-matching analyses used robust standard errors clustered by matched set. Multivariable models were adjusted for baseline and Day 7–aligned covariates as described in the Methods. The reference group was orexin receptor antagonists or ramelteon. Hazard ratios <1 indicate a lower risk of in-hospital falls in the benzodiazepines/Z-drugs group.

Prespecified subgroup analyses conducted within the matched cohort showed no evidence of effect heterogeneity across clinically relevant strata (Supplementary S3 Fig). Across all subgroups examined, hazard ratios were consistently close to unity, and all confidence intervals crossed 1.0.

## Discussion

In routine acute care for older adults who remain hospitalized beyond the first week of admission, sustained hypnotic use during hospital days 4–7 was associated with a higher incidence of in-hospital falls. This association was observed for BZ/Zs, ORA/Ram, and combination therapy compared with patients without sustained hypnotic exposure. Although the unadjusted fall risk appeared highest among patients receiving ORA/Ram, this group also exhibited greater baseline vulnerability at the landmark. After extensive confounding control using multivariable modeling with multiple imputation and a propensity score–matched comparison restricted to single-class users, fall risk did not differ significantly between BZ/Zs and ORA/Ram. The attenuation of differences after adjustment suggests that apparent variation in fall risk across hypnotic classes may primarily reflect differences in patient vulnerability and treatment selection rather than true pharmacological differences. Collectively, these findings suggest that apparent differences in fall risk across hypnotic classes observed in routine inpatient care primarily reflect patient selection and underlying vulnerability, rather than a clear class-specific pharmacologic advantage.

A key implication of our results is that ORA/Ram—despite their perceived safety profile— were not associated with lower fall risk than BZ/Zs once patient characteristics and treatment selection were rigorously accounted for. ORAs and ramelteon have mechanistic features that are often considered favorable for fall prevention, without the muscle-relaxant or ataxia-inducing effects typical of GABAergic hypnotics [16,17]. However, in routine inpatient care, prescribing is strongly influenced by clinicians’ perception of fall risk and frailty. In our cohort, ORA/Ram users were older and more functionally impaired and had greater markers of clinical complexity at the landmark, consistent with preferential prescribing to patients believed to be high-risk [18]. The substantial attenuation of crude differences after matching supports the interpretation that the elevated unadjusted risk among ORA/Ram users was driven primarily by baseline vulnerability and confounding by indication rather than by the hypnotic class itself [19,20]. In this context, adjustment for functional vulnerability is essential for valid comparison between hypnotic classes. We used the nursing care needs score as a standardized proxy for functional status at the Day 7 landmark, capturing activities of daily living, ability to understand medical or nursing instructions, and risk-related behaviors. Differences in the definition and timing of this measure compared with previous reports may partly explain discrepancies across studies.

From a clinical standpoint, changing hypnotic class alone in acute care may be insufficient as a fall-prevention strategy, given that sustained hypnotic use itself reflects heightened patient vulnerability. Notably, the absolute risk differences between exposure groups were modest (approximately 1%–3%), indicating that the clinical impact of hypnotic class selection alone may be limited. The need for sustained hypnotic treatment in hospitalized older adults may serve as a practical marker of heightened fall susceptibility, capturing unmeasured or incompletely measured factors such as prodromal delirium, nocturnal agitation, acute illness burden, pain, and environmental disorientation [21^,^^22^]. Accordingly, efforts to reduce falls may need to consider broader aspects of patient vulnerability—such as functional status, delirium risk, and the inpatient environment—rather than relying solely on pharmacologic substitution [23]. When hypnotics are used, careful monitoring may be warranted regardless of class. The inverse association observed for ICU stay should also be interpreted cautiously, as it likely reflects restricted mobility and limited ambulation in ICU settings rather than a true protective effect against falls. This may represent a form of structural bias, whereby reduced opportunity for falls contributes to the observed association, and reverse causation cannot be excluded.

Methodologically, the LM7 landmark framework strengthens the interpretability of these findings by aligning exposure assessment with the start of risk follow-up and accounting for heterogeneity in length of stay. Prior studies frequently relied on binary outcomes that ignore time at risk, potentially biasing comparisons when hospitalization duration differs between exposure groups, particularly in pharmacoepidemiologic settings [24]. By defining a pre-landmark exposure window (hospital days 4–7) and initiating follow-up on day 8 among patients who remained hospitalized and fall-free through day 7, we minimized immortal time bias and ensured a clinically coherent temporal ordering between exposure and outcome, consistent with established landmark analysis principles [25,26]. The consistency of results across multiple imputation–based adjustment, competing-risk analyses, and competing-risk sensitivity analyses further supports the robustness of our conclusions.

This study has several limitations. First, as a retrospective observational study, causal relationships between hypnotic use and in-hospital falls cannot be established, and hypnotic prescription may reflect underlying clinical vulnerability, raising the possibility of reverse causation. Second, because patients discharged within the first 7 days were excluded by design, which may preferentially remove lower-risk patients and potentially lead to overestimation of associations, our findings may not be generalizable to short-stay, lower-risk patients and apply primarily to older adults requiring prolonged hospitalization. Third, ORA/Ram may have been preferentially prescribed to patients in whom BZ/Zs were considered unsuitable. Although extensive adjustment and propensity-based analyses were performed, residual confounding due to treatment selection bias cannot be fully excluded. Finally, falls were identified through a mandatory institutional reporting system, and this study was conducted in two academic hospitals, which may limit generalizability. External validation in diverse hospital settings is warranted. Finally, the definition of hypnotic exposure in this study was based on prescription records and did not capture important details such as dosage, pharmacokinetic properties (e.g., half-life), timing and frequency of administration, whether hypnotics were used on a scheduled or as-needed basis, or the temporal relationship between drug administration and fall events. Clinically relevant factors, including bone and joint conditions (e.g., osteoarthritis or osteoporosis) and pre-admission ambulatory status, were also unavailable. In addition, hypnotics—particularly benzodiazepines—may have been prescribed for indications other than sleep (e.g., alcohol withdrawal or agitation), contributing to confounding by indication. The exposure definition based on sustained use within a fixed time window may not fully capture short-term use, discontinuation, or switching of agents. Because exposure status was fixed within the pre-landmark window, such misclassification is likely to be non-differential with respect to the outcome and may have biased the estimates toward the null. These limitations may introduce exposure heterogeneity and residual confounding. Therefore, the observed associations should not be interpreted as reflecting pure pharmacological effects but rather as associations that may also capture underlying patient characteristics and clinical decision-making. In this context, hypnotic use may also function as a proxy indicator of underlying clinical vulnerability. These findings should be interpreted as associations that may reflect underlying patient vulnerability and treatment selection rather than direct causal effects. The proportional hazards assumption was not fully satisfied, particularly for the exposure group in the multiple-imputation analyses. Therefore, the reported hazard ratios should be interpreted as average associations over the 30-day follow-up period rather than strictly time-invariant effects.

## Conclusion

In conclusion, sustained hypnotic use was associated with a higher incidence of in-hospital falls after the LM7 landmark. After accounting for clinical vulnerability and prescribing bias, no statistically significant difference in fall risk was detected between ORA/Ram and BZ/Zs. These findings indicate that substituting one hypnotic class for another alone is unlikely to reduce in-hospital falls among older adults.

## Supporting information

S1 Table Components and scoring of the institutional nursing care needs score. (DOCX)

S2 Table Distribution of individual hypnotic agents used during hospital days 4–7. (DOCX)

S3 Table Multivariable Cox proportional hazards model for in-hospital falls after the Day 7 landmark (complete-case analysis). (DOCX)

S4 Table Fine–Gray subdistribution hazards model for in-hospital falls after the Day 7 landmark (competing risk: in-hospital death). (DOCX)

S5 Table Fine–Gray subdistribution hazards model for in-hospital falls after the Day 7 landmark (competing events: in-hospital death and hospital discharge). (DOCX)

S6 Table Sensitivity analysis using categorical nursing care needs score at Day 7. (DOCX)

S1 Fig Covariate balance before and after propensity score matching between benzodiazepines/Z-drugs and ORA/Ram groups. Absolute standardized mean differences (SMDs) for baseline and Day 7–aligned covariates before and after propensity score matching are shown for patients receiving benzodiazepines/Z-drugs and those receiving orexin receptor antagonists or ramelteon. Blue circles indicate values before matching, and red triangles indicate values after matching. The vertical dashed line indicates an SMD of 0.10, representing the conventional threshold for acceptable covariate balance. After matching, covariate balance improved across most variables.
(TIFF)

S2 Fig Propensity score distributions before and after matching between benzodiazepines/Z-drugs and ORA/Ram groups. Kernel density plots show the distributions of propensity scores on the logit scale for patients receiving benzodiazepines/Z-drugs and those receiving orexin receptor antagonists or ramelteon before matching (A) and after matching (B). Red curves indicate the benzodiazepines/Z-drugs group, and blue curves indicate the ORA/Ram group. After matching, the propensity score distributions showed greater overlap between the two groups.
(TIFF)

S3 Fig Subgroup analyses comparing fall risk between benzodiazepines/Z-drugs and ORA/Ram after propensity score matching. The forest plot shows hazard ratios (HRs) and 95% confidence intervals for in-hospital falls comparing benzodiazepines/Z-drugs with orexin receptor antagonists or ramelteon across prespecified subgroups in the propensity score–matched cohort. Hazard ratios less than 1 indicate a lower fall risk in the benzodiazepines/Z-drugs group than in the ORA/Ram group. Numbers to the right of the plot indicate events and total patients in each treatment group within each subgroup. No statistically significant interaction was observed across subgroups.
(TIFF)
