# Supplementary material for: Association between hypnotic medication use and in-hospital falls among older adults: A multicenter landmark analysis
Source: PLoS One. 2026 Jun 8;21(6):e0351299. doi: 10.1371/journal.pone.0351299 (PMC13245747; doi:10.1371/journal.pone.0351299)
Supplement: S1 Table — (DOCX) [file pone.0351299.s001.docx]

**Supplementary Table S1. Components and scoring of the institutional nursing care needs score**

| Item | 0 points | 1 point | 2 points |
| --- | --- | --- | --- |
| Turning over in bed | Can do | With some help | Cannot do |
| Transfer | No assistance | Partial assistance | Full assistance |
| Oral hygiene | No assistance | Needs assistance |  |
| Food intake | No assistance | Partial assistance | Full assistance |
| Dressing/undressing | No assistance | Partial assistance | Full assistance |
| Ability to understand medical instructions /nursing instructions | Yes | No |  |
| Risky behavior | No |  | Yes |
