# Supplementary material for: Association between hypnotic medication use and in-hospital falls among older adults: A multicenter landmark analysis
Source: PLoS One. 2026 Jun 8;21(6):e0351299. doi: 10.1371/journal.pone.0351299 (PMC13245747; doi:10.1371/journal.pone.0351299)
Supplement: S2 Table — (DOCX) [file pone.0351299.s002.docx]

**Supplementary Table S2. Distribution of individual hypnotic agents used during hospital days 4–7**

| Index | Benzodiazepines / Z-drugs | No. of patients | Orexin receptor antagonists / Ramelteon | No. of patients |
| --- | --- | --- | --- | --- |
| 1 | Zolpidem tartrate | 2,363 | Lemborexant | 2,493 |
| 2 | Etizolam | 1,366 | Ramelteon | 2,399 |
| 3 | Brotizolam | 1,332 | Suvorexant | 2,330 |
| 4 | Eszopiclone | 1,001 | — | — |
| 5 | Triazolam | 548 | — | — |
| 6 | Clonazepam | 480 | — | — |
| 7 | Rilmazafone hydrochloride hydrate | 479 | — | — |
| 8 | Flunitrazepam | 377 | — | — |
| 9 | Alprazolam | 351 | — | — |
| 10 | Lorazepam | 284 | — | — |

**Footnotes:**

Hypnotic exposure was assessed during hospital days 4–7.

Patient counts represent the number of patients who received each agent on ≥1 day during this period and are not mutually exclusive, as some patients received more than one hypnotic agent.

BZ/Z indicates benzodiazepines or Z-drugs; ORA, orexin receptor antagonist.
