# Supplementary material for: Association between hypnotic medication use and in-hospital falls among older adults: A multicenter landmark analysis
Source: PLoS One. 2026 Jun 8;21(6):e0351299. doi: 10.1371/journal.pone.0351299 (PMC13245747; doi:10.1371/journal.pone.0351299)
Supplement: S3 Table — (DOCX) [file pone.0351299.s003.docx]

**Supplementary Table S3. Multivariable Cox proportional hazards model for in-hospital falls after the Day 7 landmark (complete-case analysis)**

| Variable | Multivariable HR (95% CI) | p value |
| --- | --- | --- |
| Sleep medication exposure |  |  |
| BZ/Zs only vs control | 1.616 (1.352–1.931) | <0.001 |
| ORA/Ram only vs control | 1.449 (1.195–1.756) | <0.001 |
| Combination therapy vs control | 1.707 (1.185–2.461) | 0.004 |
| Covariates (Day 7 unless noted) |  |  |
| Age (per year) | 1.010 (1.000–1.019) | 0.04 |
| Male sex | 1.138 (0.995–1.302) | 0.059 |
| Emergency admission | 0.994 (0.856–1.154) | 0.934 |
| Body mass index (kg/m²) | 0.973 (0.957–0.990) | 0.002 |
| Nursing care needs score | 1.000 (0.976–1.025) | 0.995 |
| Serum albumin (g/dL) | 0.903 (0.799–1.020) | 0.101 |
| Serum creatinine (mg/dL) | 1.030 (0.992–1.071) | 0.125 |
| Hemoglobin (g/dL) | 0.958 (0.923–0.994) | 0.023 |
| Serum sodium (mmol/L) | 0.960 (0.947–0.972) | <0.001 |
| Malignancy | 1.184 (1.021–1.372) | 0.025 |
| ICU stay (days) | 0.943 (0.914–0.973) | <0.001 |
| Oral steroids | 1.065 (0.870–1.302) | 0.543 |
| Diuretics | 1.177 (1.022–1.355) | 0.024 |
| Antiparkinsonian drugs | 1.033 (0.679–1.572) | 0.878 |
| Psychotropic drugs | 1.579 (1.340–1.862) | <0.001 |
| Antidiabetic drugs | 1.183 (1.030–1.358) | 0.018 |
| General anesthesia | 0.958 (0.763–1.201) | 0.708 |

**Footnotes:**

Hazard ratios (HRs) and 95% confidence intervals (CIs) were estimated using Cox proportional hazards models with time to first in-hospital fall after the Day 7 landmark as the outcome.

This table presents results from a complete-case analysis, restricted to patients with complete data for all covariates included in the model.

Covariates were assessed at Day 7 unless otherwise specified.

Medication exposures (oral steroids, diuretics, antiparkinsonian drugs, psychotropic drugs, antidiabetic drugs, and general anesthesia) were defined based on use during hospital days 4–7.

BZ/Zs indicates benzodiazepines or Z-drugs; ORA, orexin receptor antagonist; ICU, intensive care unit.
