# Supplementary material for: Association between hypnotic medication use and in-hospital falls among older adults: A multicenter landmark analysis
Source: PLoS One. 2026 Jun 8;21(6):e0351299. doi: 10.1371/journal.pone.0351299 (PMC13245747; doi:10.1371/journal.pone.0351299)
Supplement: S4 Table — (DOCX) [file pone.0351299.s004.docx]

**Supplementary Table S4. Fine–Gray subdistribution hazards model for in-hospital falls after the Day 7 landmark (competing risk: in-hospital death)**

| Variable | Subdistribution HR (95% CI) | p value |
| --- | --- | --- |
| Sleep medication exposure |  |  |
| BZ/Zs only vs control | 1.612 (1.347–1.929) | <0.001 |
| ORA/Ram only vs control | 1.456 (1.200–1.767) | <0.001 |
| Combination therapy vs control | 1.728 (1.200–2.489) | 0.003 |
| Covariates (Day 7 unless noted) |  |  |
| Age (per year) | 1.010 (1.001–1.019) | 0.039 |
| Male sex | 1.131 (0.989–1.293) | 0.071 |
| Emergency admission | 0.981 (0.843–1.143) | 0.81 |
| Body mass index (kg/m²) | 0.974 (0.958–0.991) | 0.002 |
| Nursing care needs score | 0.998 (0.974–1.023) | 0.891 |
| Serum albumin (g/dL) | 0.934 (0.826–1.056) | 0.274 |
| Serum creatinine (mg/dL) | 1.028 (0.990–1.068) | 0.149 |
| Hemoglobin (g/dL) | 0.955 (0.922–0.990) | 0.013 |
| Serum sodium (mmol/L) | 0.960 (0.947–0.972) | <0.001 |
| Malignancy | 1.152 (0.993–1.336) | 0.061 |
| ICU stay (days) | 0.945 (0.917–0.974) | <0.001 |
| Oral steroids | 1.041 (0.850–1.276) | 0.696 |
| Diuretics | 1.174 (1.019–1.354) | 0.027 |
| Antiparkinsonian drugs | 1.040 (0.682–1.586) | 0.857 |
| Psychotropic drugs | 1.570 (1.326–1.858) | <0.001 |
| Antidiabetic drugs | 1.173 (1.020–1.348) | 0.026 |
| General anesthesia | 0.978 (0.781–1.224) | 0.846 |

**Footnotes:**Subdistribution hazard ratios (sHRs) and 95% confidence intervals (CIs) were estimated using Fine–Gray subdistribution hazards models, treating in-hospital death as a competing event.
The outcome was time to first in-hospital fall after the Day 7 landmark.
Covariates were assessed at Day 7 unless otherwise specified.
Medication exposures were defined based on use during hospital days 4–7.
BZ/Zs indicates benzodiazepines or Z-drugs; ORA, orexin receptor antagonist; ICU, intensive care unit.
