# Supplementary material for: Association between hypnotic medication use and in-hospital falls among older adults: A multicenter landmark analysis
Source: PLoS One. 2026 Jun 8;21(6):e0351299. doi: 10.1371/journal.pone.0351299 (PMC13245747; doi:10.1371/journal.pone.0351299)
Supplement: S5 Table — (DOCX) [file pone.0351299.s005.docx]

**Supplementary Table S5. Fine–Gray subdistribution hazards model for in-hospital falls after the Day 7 landmark (competing events: in-hospital death and hospital discharge)**

| Variable | Subdistribution HR (95% CI) | p value |
| --- | --- | --- |
| Sleep medication exposure |  |  |
| BZ/Zs only vs control | 1.655 (1.384–1.980) | <0.001 |
| ORA/Ram only vs control | 1.503 (1.237–1.827) | <0.001 |
| Combination therapy vs control | 1.768 (1.227–2.546) | 0.002 |
| Covariates (Day 7 unless noted) |  |  |
| Age (per year) | 1.005 (0.996–1.014) | 0.281 |
| Male sex | 1.147 (1.003–1.310) | 0.044 |
| Emergency admission | 1.109 (0.949–1.295) | 0.192 |
| Body mass index (kg/m²) | 0.974 (0.957–0.990) | 0.002 |
| Nursing care needs score | 1.060 (1.035–1.086) | <0.001 |
| Serum albumin (g/dL) | 0.748 (0.659–0.849) | <0.001 |
| Serum creatinine (mg/dL) | 1.042 (1.005–1.081) | 0.027 |
| Hemoglobin (g/dL) | 0.937 (0.902–0.973) | <0.001 |
| Serum sodium (mmol/L) | 0.950 (0.937–0.963) | <0.001 |
| Malignancy | 1.216 (1.048–1.411) | 0.01 |
| ICU stay (days) | 0.939 (0.911–0.969) | <0.001 |
| Oral steroids | 1.203 (0.983–1.472) | 0.073 |
| Diuretics | 1.229 (1.068–1.414) | 0.004 |
| Antiparkinsonian drugs | 1.034 (0.678–1.577) | 0.877 |
| Psychotropic drugs | 1.594 (1.343–1.892) | <0.001 |
| Antidiabetic drugs | 1.237 (1.075–1.424) | 0.003 |
| General anesthesia | 1.096 (0.873–1.374) | 0.43 |

Footnotes:

Subdistribution hazard ratios (sHRs) and 95% confidence intervals (CIs) were estimated using Fine–Gray subdistribution hazards models, treating in-hospital death and hospital discharge as competing events.

The outcome was time to first in-hospital fall after the Day 7 landmark.

Covariates were assessed at Day 7 unless otherwise specified.

Medication exposures were defined based on use during hospital days 4–7.

BZ/Zs indicates benzodiazepines or Z-drugs; ORA, orexin receptor antagonist; ICU, intensive care unit.
