# Supplementary material for: Association between hypnotic medication use and in-hospital falls among older adults: A multicenter landmark analysis
Source: PLoS One. 2026 Jun 8;21(6):e0351299. doi: 10.1371/journal.pone.0351299 (PMC13245747; doi:10.1371/journal.pone.0351299)
Supplement: S6 Table — (DOCX) [file pone.0351299.s006.docx]

**Supplementary Table S6. Sensitivity analysis using categorical nursing care needs score at Day 7**

| Variable | Complete-case HR (95% CI) | p value | Multiple imputation HR (95% CI) | p value |
| --- | --- | --- | --- | --- |
| Sleep medication exposure |  |  |  |  |
| BZ/Zs only vs control | 1.619 (1.355–1.935) | <0.001 | 1.424 (1.237–1.640) | <0.001 |
| ORA/Ram only vs control | 1.442 (1.190–1.748) | <0.001 | 1.454 (1.250–1.692) | <0.001 |
| Combination therapy vs control | 1.710 (1.187–2.465) | 0.004 | 1.412 (1.047–1.906) | 0.024 |
| Covariates (Day 7 unless noted) |  |  |  |  |
| Age (per year) | 1.009 (1.000–1.019) | 0.05 | 1.008 (1.001–1.016) | 0.022 |
| Male sex | 1.140 (0.997–1.304) | 0.056 | 1.188 (1.069–1.320) | 0.001 |
| Emergency admission | 0.989 (0.851–1.149) | 0.884 | 0.974 (0.868–1.092) | 0.650 |
| Body mass index (kg/m²) | 0.973 (0.957–0.990) | 0.002 | 0.974 (0.961–0.988) | <0.001 |
| Nursing care needs score (categorical) |  |  |  |  |
| Middle vs Low | 0.978 (0.816–1.173) | 0.812 | 1.011 (0.882–1.160) | 0.873 |
| High vs Low | 1.038 (0.848–1.270) | 0.719 | 1.036 (0.886–1.210) | 0.660 |
| Serum albumin (g/dL) | 0.908 (0.803–1.027) | 0.124 | 0.894 (0.796–1.004) | 0.058 |
| Serum creatinine (mg/dL) | 1.031 (0.992–1.071) | 0.122 | 1.028 (0.990–1.067) | 0.152 |
| Hemoglobin (g/dL) | 0.957 (0.922–0.993) | 0.021 | 0.957 (0.925–0.990) | 0.011 |
| Serum sodium (mmol/L) | 0.959 (0.947–0.972) | <0.001 | 0.959 (0.948–0.971) | <0.001 |
| Malignancy | 1.190 (1.027–1.378) | 0.021 | 1.244 (1.111–1.394) | <0.001 |
| ICU stay (days) | 0.940 (0.912–0.970) | <0.001 | 0.952 (0.927–0.977) | <0.001 |
| Oral steroids | 1.065 (0.871–1.303) | 0.538 | 1.192 (1.028–1.381) | 0.020 |
| Diuretics | 1.178 (1.024–1.357) | 0.022 | 1.145 (1.023–1.281) | 0.018 |
| Antiparkinsonian drugs | 1.024 (0.673–1.556) | 0.913 | 1.245 (0.940–1.651) | 0.127 |
| Psychotropic drugs | 1.566 (1.330–1.843) | <0.001 | 1.621 (1.428–1.840) | <0.001 |
| Antidiabetic drugs | 1.181 (1.029–1.357) | 0.018 | 1.125 (1.007–1.256) | 0.037 |
| General anesthesia | 0.956 (0.761–1.201) | 0.699 | 0.918 (0.751–1.123) | 0.407 |

**Footnotes:**

Hazard ratios (HRs) and 95% confidence intervals (CIs) were estimated using Cox proportional hazards models with time to first in-hospital fall after the Day 7 landmark as the outcome.
This table presents results from a complete-case analysis, restricted to patients with complete data for all covariates included in the model.
Covariates were assessed at Day 7 unless otherwise specified.
Medication exposures (oral steroids, diuretics, antiparkinsonian drugs, psychotropic drugs, antidiabetic drugs, and general anesthesia) were defined based on use during hospital days 4–7.
The nursing care needs score at Day 7 was categorized into three groups: Low (score = 0, reference), Middle (score = 1–4), and High (score ≥5).
BZ/Zs indicates benzodiazepines or Z-drugs; ORA, orexin receptor antagonist; ICU, intensive care unit.
