# Supplementary figures and images for: Association between hypnotic medication use and in-hospital falls among older adults: A multicenter landmark analysis
Source: PLoS One. 2026 Jun 8;21(6):e0351299. doi: 10.1371/journal.pone.0351299 (PMC13245747; doi:10.1371/journal.pone.0351299)

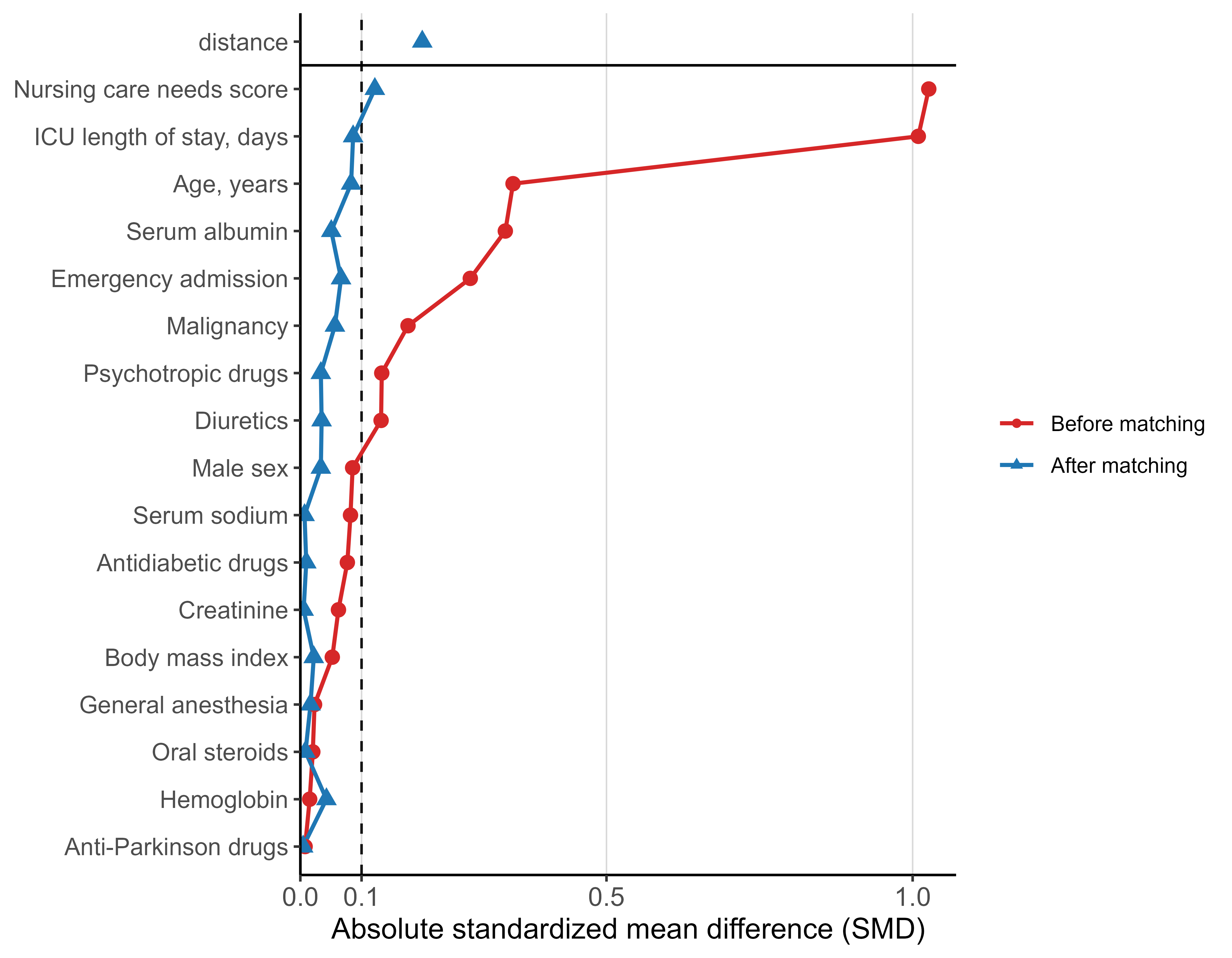

Supplement: S1 Fig — Absolute standardized mean differences (SMDs) for baseline and Day 7–aligned covariates before and after propensity score matching are shown for patients receiving benzodiazepines/Z-drugs and those receiving orexin receptor antagonists or ramelteon. Blue circles indicate values before matching, and red triangles indicate values after matching. The vertical dashed line indicates an SMD of 0.10, representing the conventional threshold for acceptable covariate balance. After matching, covariate balance improved across most variables. (TIFF) [file pone.0351299.s007.tiff]

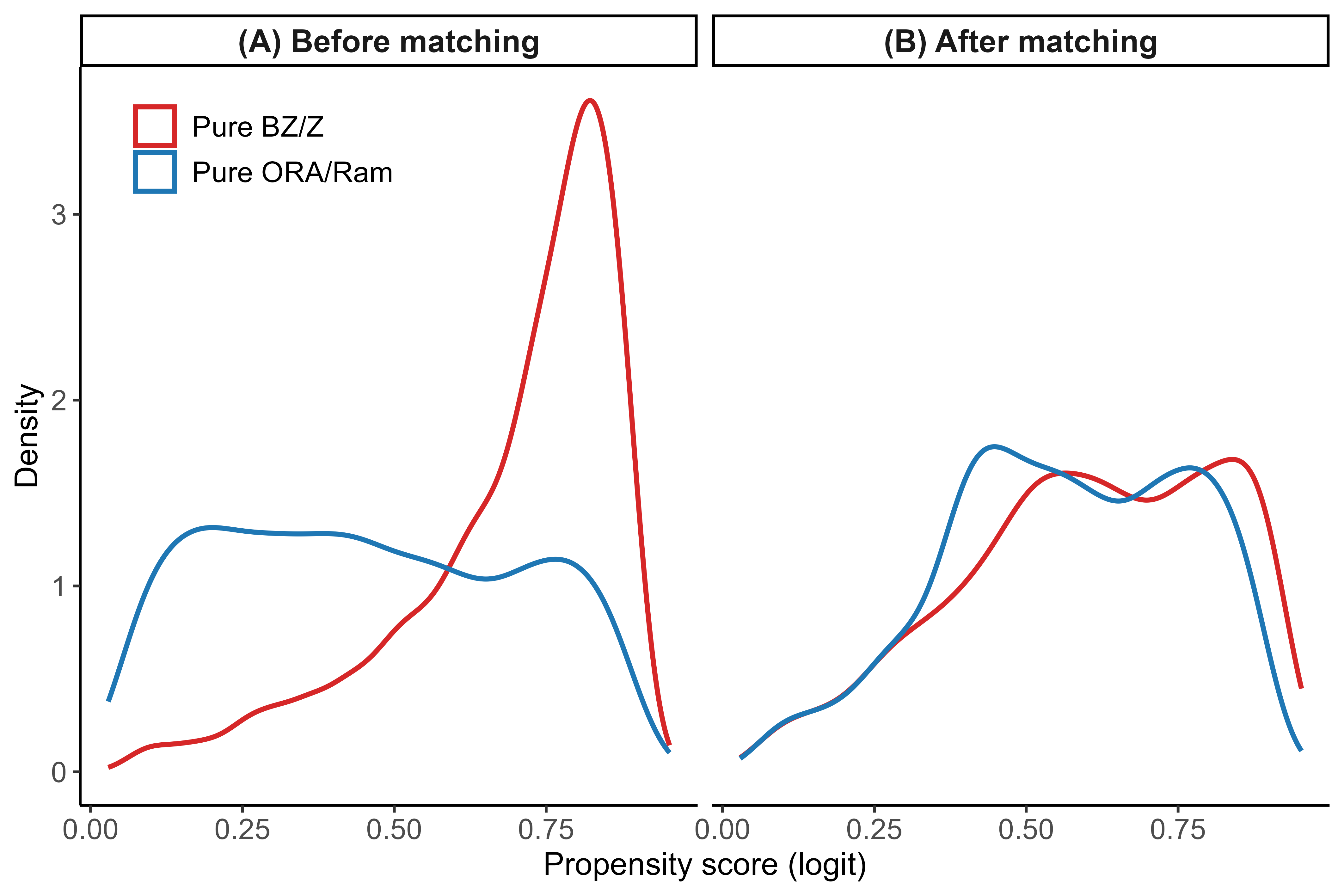

Supplement: S2 Fig — Kernel density plots show the distributions of propensity scores on the logit scale for patients receiving benzodiazepines/Z-drugs and those receiving orexin receptor antagonists or ramelteon before matching (A) and after matching (B). Red curves indicate the benzodiazepines/Z-drugs group, and blue curves indicate the ORA/Ram group. After matching, the propensity score distributions showed greater overlap between the two groups. (TIFF) [file pone.0351299.s008.tiff]

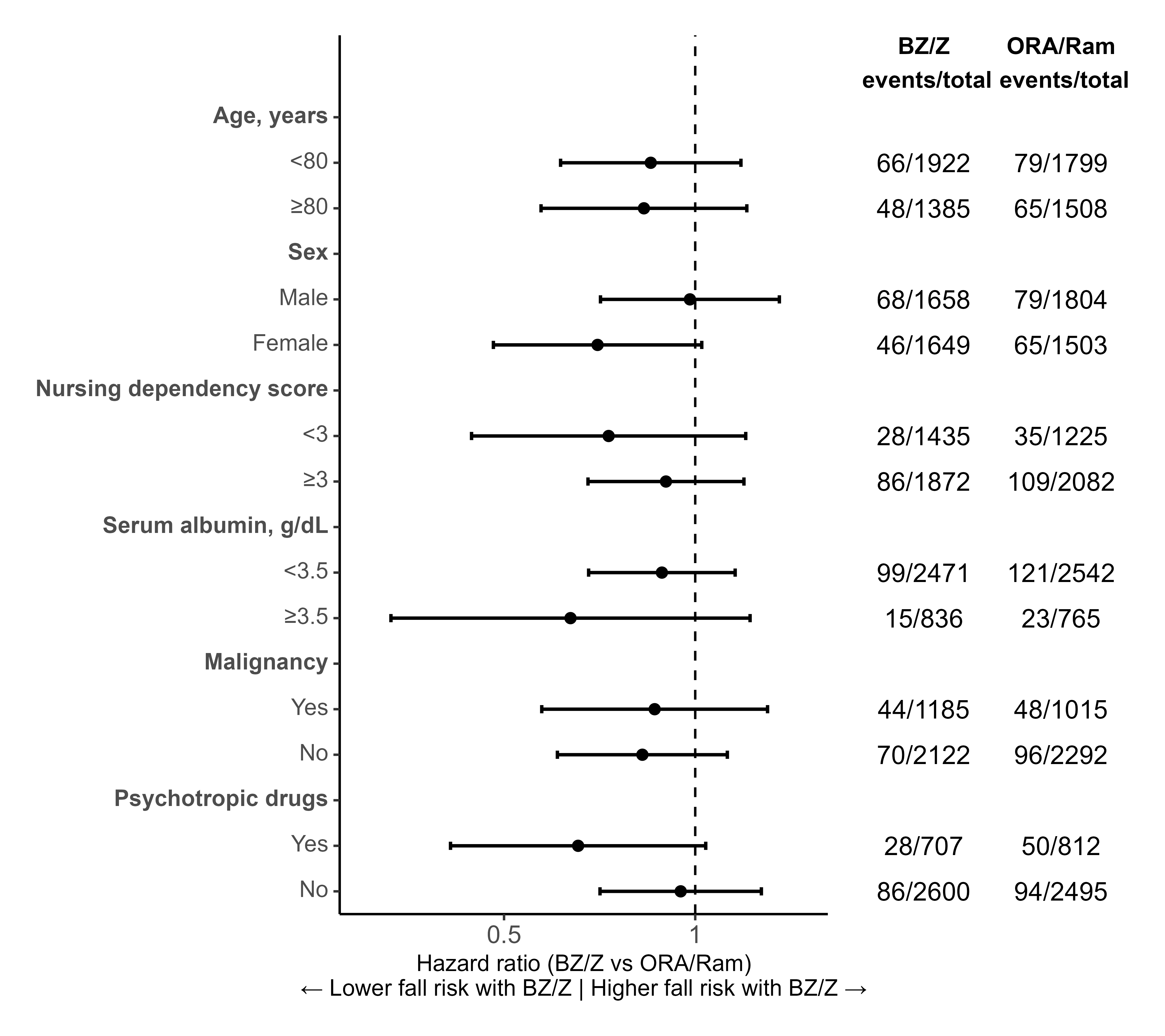

Supplement: S3 Fig — The forest plot shows hazard ratios (HRs) and 95% confidence intervals for in-hospital falls comparing benzodiazepines/Z-drugs with orexin receptor antagonists or ramelteon across prespecified subgroups in the propensity score–matched cohort. Hazard ratios less than 1 indicate a lower fall risk in the benzodiazepines/Z-drugs group than in the ORA/Ram group. Numbers to the right of the plot indicate events and total patients in each treatment group within each subgroup. No statistically significant interaction was observed across subgroups. (TIFF) [file pone.0351299.s009.tiff]
